# A combination of two variants in *PRKAG3* is needed for a positive effect on meat quality in pigs

**DOI:** 10.1186/1471-2156-15-29

**Published:** 2014-02-28

**Authors:** Pekka Uimari, Anu Sironen

**Affiliations:** 1 MTT Agrifood Research Finland, Biotechnology and Food Research, FI-31600 Jokioinen, Finland; 2 Department of Agricultural Sciences, Animal Breeding, University of Helsinki, FI-00014 Helsinki, Finland

**Keywords:** Association, Haplotype, Meat quality, Pig, SNP

## Abstract

**Background:**

Color and pH of meat measured 24 h post mortem are common selection objectives in pig breeding programs. Several amino acid substitutions in *PRKAG3* have been associated with various meat quality traits. In our previous study ASGA0070625, a SNP next to *PRKAG3*, had the most significant association with meat quality traits in the Finnish Yorkshire. However, the known amino acid substitutions, including I199V, did not show any association. The aims of this study were to characterize further variation in *PRKAG3* and its promoter region, and to test the association between these variants and the pH and color of pork meat.

**Results:**

The data comprised of 220 Finnish Landrace and 230 Finnish Yorkshire artificial insemination boars with progeny information. We sequenced the coding and promoter region of *PRKAG3* in these and in three additional wild boars. Genotypes from our previous genome-wide scans were also included in the data. Association between SNPs or haplotypes and meat quality traits (deregressed estimates of breeding values from Finnish national breeding value estimation for pH, color lightness and redness measured from loin or ham) was tested using a linear regression model. Sequencing revealed several novel amino acid substitutions in *PRKAG3*, including K24E, I41V, K131R, and P134L. Linkage disequilibrium was strong among the novel variants, SNPs in the promoter region and ASGA0070625, especially for the Yorkshire. The strongest associations were observed between ASGA0070625 and the SNPs in the promoter region and pH measured from loin in the Yorkshire and between I199V and pH measured from ham in the Landrace. In contrast, ASGA0070625 was not significantly associated with meat quality traits in the Landrace and I199V not in the Yorkshire. Haplotype analysis showed a significant association between a haplotype consisting of *199I* and *24E* alleles (or *g.-157C* or *g.-58A* alleles in the promoter region) and pH measured from loin and ham in both breeds (P-values varied from 1.72 × 10^-4^ to 1.80 × 10^-8^).

**Conclusions:**

We conclude that haplotype *g.-157C - g.-58A - 24E - 199I* in *PRKAG3* has a positive effect on meat quality in pigs. Our results are readily applicable for marker-assisted selection in pigs.

## Background

Meat quality characteristics such as water-holding capacity, tenderness, intramuscular fat, and taste are important for the meat industry as well as for consumers [1-3]. Since direct measurement of some of these traits is considered laborious in practice, many breeding programs only use correlated traits like pH and color of meat for selection. In Finland, pH and color of loin have been among the pig breeding objectives since 1983 and meat quality of ham since 2000 [1].

Several important genes are known to have a major effect on meat quality and carcass composition traits in pigs, including *RYR1* (ryanodine receptor 1) on chromosome 6 [4]; *PRKAG3* (protein kinase, AMP-activated, gamma 3 non-catalytic subunit) on chromosome 15 [5,6]; *IGF2* (insulin-like growth factor 2) on chromosome 2 [7,8]; *CAST* (calpastatin) on chromosome 2 [9]; and *MC4R* (melanocortin 4 receptor) on chromosome 1 [10]. A previous genome-wide association study in the Finnish Yorkshire population revealed a strong association between pH measured from loin and a chromosomal region around *PRKAG3*[11]. However, none of the reported amino acid substitutions T30N, G52S, L53P, I199V, or R200Q were as strongly associated with pH as SNP ASGA0070625 (RS80816788, position 133,677,385 Sus Scrofa build 10.2), which is located near the *PRKAG3* gene. A further study with more markers on the *PRKAG3* region was therefore needed. Additionally, the Finnish Landrace breed could be used as a validation population for the initial findings.

This article reports novel variations within the *PRKAG3* gene in Finnish Yorkshire and Landrace pigs, shows the linkage disequilibrium (LD) structure around *PRKAG3*, and presents the results from our association analysis between SNPs or haplotypes and meat quality traits. We show that to achieve a positive effect on meat quality traits, particularly in pH measured post mortem from loin and ham, the animal has to carry both the *199I* allele and the combination *g.-157C - g.-58A - 24E*. This haplotype was significant for meat quality in both breeds.

## Results

### SNP discovery

Sequencing of the exons and promoter region of *PRKAG3* revealed four novel amino acid substitutions: K24E, I41V, K131R, and P134L (Table 1). The four SNPs upstream of the *PRKAG3* transcription initiation site reported by Ryan et al. [12] were also detected in the studied Finnish Yorkshire and Landrace populations. Two additional synonymous substitutions were observed at amino acids 193 and 194, but these were very rare in both populations (frequency less than 3%) and were thus excluded from later analysis. Minor allele frequencies of the novel amino acid substitutions varied from 0.17 to 0.20 and from 0.15 to 0.20 in the Yorkshire and Landrace, respectively. Alleles *53P* and *200R* were fixed in both populations. Allele *30T* was also very rare in the Yorkshire, with a frequency of only 1% compared to 13% in the Landrace, whereas alleles *52S* and *199I* were more common in the Yorkshire than in the Landrace.

**Table 1:** **Positions, alleles and minor allele frequencies of the identified SNPs in *PRKAG3***

**Exon**	**Variation**^**a**^	**SS number**	**RS number**	**Position on chromosome 15, bp**^**b**^	**Alleles**^**c**^	**Yorkshire**	**Landrace**
	ASGA0070625		80816788	133677385	A/G	0.21	0.28
	g.-311A > G		196959880	133800456	G/A	0.19	0.13
	g.-221G > A		196952335	133800546	A/G	0.19	0.13
	g.-157C > G		196956394	133800610	G/C	0.20	0.26
	g.-58A > G		196959698	133800709	G/A	0.20	0.26
2	***K24E***	947848631		133801207	A/G	0.17	0.20
3	T30N		328566929	133802071	A/C	0.01	0.13
3	***I41V***	947848632		133802103	A/G	0.20	0.16
3	G52S		343733804	133802136	A/G	0.43	0.12
4	L53P		337462352	133802499	T/C	0	0
4	***K131R***	947848633		133802733	G/A	0.19	0.15
4	***P134L***	947848634		133802742	T/C	0.19	0.16
5	I199V			133803828	A/G	0.45	0.30
5	R200Q			133803829	A/G	0	0

^a^The novel amino acid substitutions are given in italics with corresponding dbSNP submission numbers (http://www.ncbi.nlm.nih.gov/SNP/).

^b^Based on Sus Scrofa build 10.2.

^c^The minor allele is given first.

Functional analysis of the amino acid substitutions using SIFT [13] (Sorting Intolerant From Tolerant, http://sift.bii.a-star.edu.sg/) underlined three substitutions, K24E, I41V and L53P, as damaging (Table 2). These effects appeared to be transcript specific indicating differences between effects of substitutions on protein isoforms. For substitutions I41V and L53P the SIFT scores were similar for all protein isoforms, but a clear difference for K24E was identified (Table 2).

**Table 2:** **Effect of the identified SNPs in *PRKAG3 *** **on protein sequence**

**Reference**	**Position on chromosome 15, bp**	**Amino acid co-ordinate**	**Transcript**	**SIFT**^**a**^
K24E	133801207	47	ENSSSCT00000017641	0.16
K24E	133801207	24	ENSSSCT00000033825	0.01
T30N	133802071	80	ENSSSCT00000017641	0.44
T30N	133802071	57	ENSSSCT00000033825	0.20
T30N	133802071	30	ENSSSCT00000036402	0.32
I41V	133802103	91	ENSSSCT00000017641	0.05
I41V	133802103	68	ENSSSCT00000033825	0.07
I41V	133802103	41	ENSSSCT00000036402	0.03
G52S	133802136	102	ENSSSCT00000017641	0.11
G52S	133802136	79	ENSSSCT00000033825	0.13
G52S	133802136	52	ENSSSCT00000036402	0.13
L53P	133802499	103	ENSSSCT00000017641	0.04
L53P	133802499	80	ENSSSCT00000033825	0.05
L53P	133802499	53	ENSSSCT00000036402	0.07
K131R	133802733	181	ENSSSCT00000017641	1.00
K131R	133802733	131	ENSSSCT00000036402	1.00
K131R	133802733	158	ENSSSCT00000033825	1.00
P134L	133802742	184	ENSSSCT00000017641	0.58
P134L	133802742	134	ENSSSCT00000036402	0.58
P134L	133802742	161	ENSSSCT00000033825	0.58
I199V	133803828	249	ENSSSCT00000017641	1.00
I199V	133803828	199	ENSSSCT00000036402	1.00
I199V	133803828	226	ENSSSCT00000033825	1.00

^a^SIFT score <0.05 is considered as damaging.

### Linkage disequilibrium

**Figure 1:**
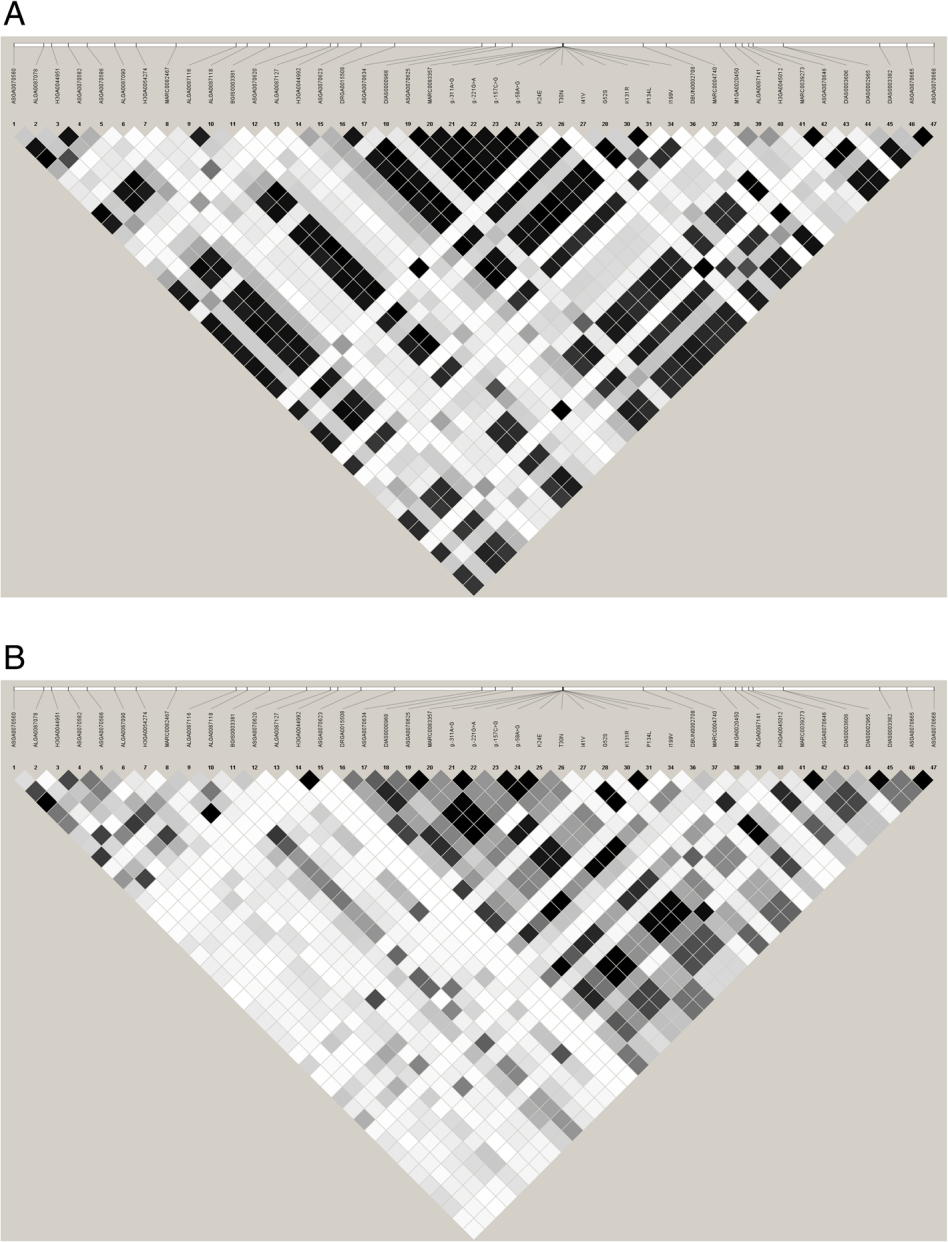
**Linkage disequilibrium (LD) expressed as r**^**2 **^**values (black color indicated complete LD) within and around *PRKAG3 *** **for the Finnish Yorkshire (A) and Landrace (B).**

Figure 1 shows the linkage disequilibrium (LD) within and around *PRKAG3*. Overall, LD was stronger and extended over a longer range in the Finnish Yorkshire than Landrace. In the Yorkshire, g.-311A > G, g.-221G > A, g.-157C > G, g.-58A > G, K24E, I41V, K131R, and P134L were in complete LD with ASGA0070625, the SNP that showed the strongest association with pH measured from loin in our previous whole-genome analysis [11]. In the Finnish Landrace, ASGA0070625 was in complete LD with g.-157C > G, g.-58A > G, and K24E. Interestingly, the known amino acid substitutions T30N, G53S, and I199V were in very weak LD with ASGA0070625 and, hence, also with the novel amino acid substitutions and SNPs in the promoter region of *PRKAG3* in both breeds.

### Haplotypes

In total, 12 different haplotypes were observed in either of the breeds (Table 3). One of these, HAP3, is actually the same as the wild haplotype identified in the sequences from the European wild boars used in this study (based on three wild boar samples). HAP3 was the most common haplotype in the Finnish Landrace breed (frequency 0.34), but very rare in the Finnish Yorkshire (frequency 0.01). HAP1, a haplotype similar to the wild haplotype except for a point mutation of G to A at G52S, was the most frequent in the Yorkshire with a frequency of 0.42. Again there was a notable difference in frequencies between the two breeds (frequency only 0.12 in the Landrace). HAP2, another haplotype similar to the wild haplotype except for a point mutation of G to A at I199V, was common in both breeds, with frequencies of 0.36 and 0.25 for the Yorkshire and Landrace, respectively. The other haplotypes (HAP4 to HAP12) jointly accounted for 22% to 29% of the haplotypes identified in the Yorkshire and Landrace, respectively. Some of the rare haplotypes may be spurious due to genotyping or haplotyping errors.

**Table 3:** **Haplotypes in *PRKAG3 *** **and their frequencies**

	**1**	**2**	**3**	**4**	**5**	**6**	**7**	**8**	**9**	**10**	**11**	**12**	**Wild boar**
ASGA0070625	G	G	G	G	A	A	A	A	A	A	A	A	
g.-311A > G	A	A	A	A	A	A	A	G	G	G	G	G	A
g.-221G > A	G	G	G	G	G	G	G	A	A	A	A	A	G
g.-157C > G	C	C	C	C	G	G	G	G	G	G	G	G	C
g.-58A > G	A	A	A	A	G	G	G	G	G	G	G	G	A
K24E	G	G	G	G	A	G	G	A	A	A	A	A	G
T30N	C	C	C	C	A	A	A	C	C	C	C	A	C
I41V	G	G	G	G	G	G	G	A	A	A	G	G	G
G52S	A	G	G	A	G	G	G	G	G	G	G	G	G
K131R	A	A	A	A	A	A	A	G	G	G	A	A	A
P134L	C	C	C	C	C	C	C	T	T	T	C	C	C
I199V	G	A	G	A	G	A	G	A	G	G	G	G	G
Yorkshire	0.418	0.363	0.007	0.007	0.002	0.002	0.007	0.080	0.087	0.027	0	0	
Landrace	0.122	0.249	0.341	0	0.124	0	0	0.041	0.116	0	0.002	0.004	

### SNP association

**Figure 2:**
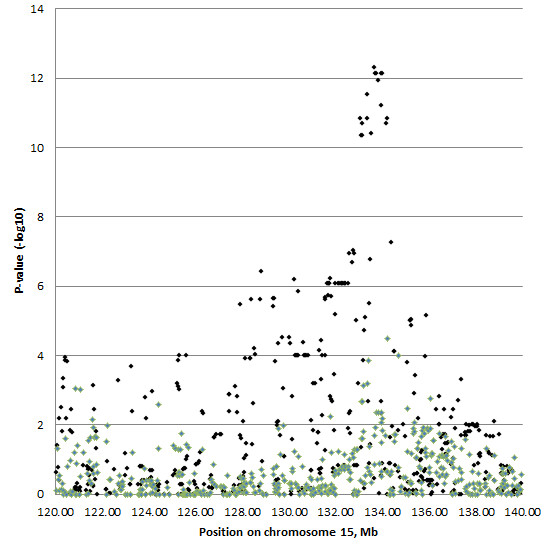
**P-values (-log10) of the SNPs for pH measured from loin.** P-values for the Finnish Yorkshire are marked with black diamonds and for the Finnish Landrace with green diamonds.

Based on the genotypes from our previous whole-genome scans [11,14], the most significant SNPs affecting meat quality traits were in the region from 120 Mb to 140 Mb on chromosome 15 in the proximity of *PRKAG3* (Figure 2). The smallest P-value was observed for association between six SNPs (ASGA0070634, ASGA0070625, MARC0083357, DBUN0002708, MARC0039273, and DIAS0002965) and pH measured from loin in the Finnish Yorkshire. These six SNPs were in complete LD in the region from 133.64 Mb to 134.01 Mb. Their estimated allele substitution effect for the Yorkshire was -0.059 ± 0.008 (P-value = 7.28 × 10^-13^), corresponding to 1.3 SD of the estimated polygenic effect. Minor alleles *A, A, C, A, A*, and *G* of ASGA0070634, ASGA0070625, MARC0083357, DBUN0002708, MARC0039273, and DIAS0002965, respectively, decreased pH measured from loin. None of these six SNPs showed statistically significant association with any of the measured traits in the Finnish Landrace (see Figure 2 for pH measured from loin, as an example). Statistical significance was claimed if the P-value was below 2.0 × 10^-6^. This stringent limit was set because the observations used in this study were the same as those in our previous genome-wide scans [11,14].

Because of the strong LD with ASGA0070625, the novel amino acid substitutions identified in this study and SNPs in the promoter region of *PRKAG3* gave very small P-values for pH measured from loin for the Finnish Yorkshire (Table 4). The differences in P-values between ASGA0070625 and the SNPs which were in complete LD with ASGA0070625 were due to slight differences in the number of genotypes available for each SNP. No significant association was detected between I199V and the measured meat quality traits for the Yorkshire. For the Landrace, in contrast, the strongest association was observed between I199V and pH measured from ham (P-value = 6.44 × 10^-7^ with an allele substitution effect of 0.030 ± 0.006). The association between I199V and pH measured from loin was also strong in the Landrace (Table 4).

**Table 4:** **P-values of the association between SNPs in *PRKAG3 *** **and meat quality traits**

	**Yorkshire**						**Landrace**					
	**Loin**			**Ham**			**Loin**			**Ham**		
**Variation**	**pH**	L*	a*	**pH**	L*	a*	**pH**	L*	a*	**pH**	L*	a*
ASGA0070625	7.27 × 10^-13^	5.52 × 10^-6^	4.38 × 10^-5^	3.47 × 10^-6^	0.73	8.85 × 10^-3^	0.02	0.03	0.03	0.02	0.34	0.79
g.-311A > G	6.83 × 10^-10^	4.22 × 10^-5^	6.28 × 10^-4^	1.44 × 10^-4^	0.74	0.05	0.28	0.36	0.36	0.60	0.93	0.79
g.-221G > A	6.83 × 10^-10^	4.22 × 10^-5^	6.28 × 10^-4^	1.44 × 10^-4^	0.74	0.05	0.33	0.62	0.36	0.64	0.98	0.77
g.-157C > G	7.94 × 10^-11^	2.79 × 10^-5^	4.29 × 10^-4^	2.68 × 10^-5^	0.56	0.03	0.02	0.06	0.37	0.05	0.38	0.83
g.-58A > G	7.94 × 10^-11^	2.79 × 10^-5^	4.29 × 10^-4^	2.68 × 10^-5^	0.56	0.03	0.03	0.09	0.36	0.06	0.41	0.92
K24E	5.80 × 10^-10^	3.83 × 10^-5^	3.16 × 10^-3^	7.83 × 10^-5^	0.38	0.03	0.23	0.27	0.16	0.15	0.57	0.54
T30N	0.13	0.39	0.46	0.12	0.61	0.36	0.02	6.51 × 10^-3^	0.32	0.01	0.16	0.50
I41V	1.41 × 10^-11^	1.43 × 10^-5^	9.20 × 10^-5^	1.96 × 10^-5^	0.82	0.02	0.45	0.92	0.04	0.68	0.53	0.59
G52S	0.36	0.58	0.21	0.77	0.91	0.81	0.41	0.79	0.84	0.78	0.53	0.64
K131R	1.41 × 10^-11^	1.43 × 10^-5^	9.20 × 10^-5^	1.96 × 10^-5^	0.82	0.02	0.41	0.93	0.08	0.54	0.60	0.62
P134L	1.41 × 10^-11^	1.43 × 10^-5^	9.20 × 10^-5^	1.96 × 10^-5^	0.82	0.02	0.43	0.85	0.04	0.70	0.55	0.67
I199V	0.01	0.19	0.12	0.09	0.93	0.10	1.62 × 10^-5^	0.09	0.04	6.44 × 10^-7^	0.17	0.67
DBUN0002708	3.21 × 10^-10^	4.15 × 10^-5^	3.81 × 10^-4^	3.36 × 10^-4^	0.87	0.01	5.37 × 10^-3^	0.02	0.05	0.07	0.37	0.75

### Haplotype association

Four haplotypes were identified having allele *G* of ASGA0070625 (Table 3). When each of these was tested against all other haplotypes in the Yorkshire data, the only statistically significant association (P-value = 6.35 × 10^-8^, Table 5) was observed between HAP2 (with *199I* or allele *A*) and pH measured from loin. The substitution effect of this haplotype was 0.039 ± 0.007. HAP2 was also the only haplotype that showed a significant association in the Finnish Landrace with both pH measurements (Table 5). The haplotype substitution effects for pH measured from loin and ham were 0.031 ± 0.007 and 0.039 ± 0.007, respectively. Haplotypes carrying allele *A* of ASGA0070625 (HAP5-HAP12) had no significant association with any of the tested meat quality traits. Haplotypes with only one or two observations (HAP6, HAP11, and HAP12) were excluded from the analysis.

**Table 5:** **P-values of the associations between haplotypes in *PRKAG3 *** **and meat quality traits**

	**Yorkshire**						**Landrace**					
	**Loin**			**Ham**			**Loin**			**Ham**		
**Haplotype**^**a**^	**pH**	L*	a*	**pH**	L*	a*	**pH**	L*	a*	**pH**	L*	a*
HAP1	0.47	0.90	0.14	0.76	0.69	0.66	0.41	0.89	0.92	0.65	0.61	0.57
HAP2	6.35 × 10^-8^	6.00 × 10^-4^	0.01	1.72 × 10^-4^	0.97	0.01	8.69 × 10^-6^	0.15	3.99 × 10^-3^	1.80 × 10^-8^	0.35	0.41
HAP3	0.15	0.51	0.17	0.40	0.03	5.98 × 10^-3^	0.04	0.41	0.17	0.02	0.74	0.13
HAP4	0.42	0.06	0.24	0.88	0.23	0.38						
HAP5							0.02	0.01	0.27	0.02	0.17	0.60
HAP7	0.73	0.44	0.78	0.16	0.80	0.32						
HAP8	2.25 × 10^-4^	3.24 × 10^-3^	0.11	3.03 × 10^-3^	0.82	0.35	0.12	0.05	0.83	0.12	0.08	0.48
HAP9	2.29 × 10^-4^	0.05	4.98 × 10^-4^	0.13	0.48	0.08	0.07	0.23	0.12	0.16	0.63	0.94
HAP10	0.04	0.17	0.50	0.11	0.38	0.42						

^a^Because of low number of observations, haplotypes HAP6, HAP11, and HAP12 were excluded from the analysis.

## Discussion

Meat color and pH are traits which are commonly included in pig breeding programs to improve the technological properties of pork and to increase consumer gratification. Several studies indicate that *PRKAG3* is one of the key genes causing variation in pork meat pH, L* (lightness of color), and drip loss between animals [5,6,15-18]. The protein encoded by *PRKAG3* is the skeletal muscle cell-specific regulatory subunit gamma 3 of AMP-activated protein kinase (AMPK). AMPK is an energy sensor which, when activated in response to cellular metabolic stresses, directly phosphorylates and inactivates the key enzymes involved in regulating de novo biosynthesis of fatty acid and cholesterol. The best known mutation in *PRKAG3* is *200Q*, which is found only in the Hampshire pig breed. The allele *200Q* causes a high content of stored glycogen in white skeletal muscles, leading to low muscle pH 24 h post mortem, poor water-holding capacity, and low processing yield [5]. Furthermore, I199V and T30N are reported to affect pH [6,15,17,18], and variations in the promoter region of *PRKAG3* have been associated with gene expression and meat quality [12].

In this study, we characterized several novel amino acid substitutions within exons 2, 3, and 4. The SNPs characterized in the *PRKAG3* promoter region correspond to those reported by Ryan et al. [12]. Based on the genomic sequence the SNP g.-158C > G reported by Ryan et. al. corresponds to our g.-157C > G. The genomic location of these SNPs is the same. All novel amino acid substitutions (K24E, I41V, K131R, and P134L) and characterized SNPs in the promoter region were in complete LD in the Finnish Yorkshire, based on Haploview analysis. The Finnish Landrace breed showed more diversity, with only g.-157C > G, g.-58A > G, and K24E in complete LD with each other and with ASGA0070625. Strong but not complete LD was also reported by Ryan et al. [12] for SNPs in the promoter region. No significant LD was found between the promoter region SNPs or novel amino acid substitutions and I199V. This finding is similar to the observation made by Ryan et al. [12] regarding LD between promoter region SNPs and I199V.

A slightly different picture of LD can be drawn from the haplotype estimates obtained by the FastPHASE program (Table 3). Haplotypes HAP5, HAP6, and HAP7 divide the LD pattern into two groups. The first group comprises the SNPs in complete LD, namely ASGA0070625, g.-157C > G, and g.-58A > G in the Finnish Yorkshire, and additionally K24E in Finnish Landrace. For Landrace, this is exactly the same result as given by Haploview. The second group includes SNPs g.-311A > G, g.-221G > A, I41V, K131R, and P134L in the Yorkshire, but not in the Landrace.

There are several explanations for the different LD outcomes from Haploview and FastPHASE analyses, such as different algorithms and ways of treating missing genotypes between the two programs. Genotyping or phasing errors may also cause spurious haplotypes. The latter view is supported by the fact that haplotypes HAP6 and HAP7 are extremely rare in the Yorkshire and absent in the Landrace, while haplotypes HAP11 and HAP12 are completely absent in the Yorkshire and are carried only by one or two animals in Landrace. Additionally, some haplotypes may have been introduced into one breed from the other through occasional involuntary crossing of breeds at the farm level.

Single-SNP analysis yielded controversial results when the two breeds were compared. The SNP which was reported as highly significant for pH measured from loin in the Finnish Yorkshire in our previous study [11] was significant also in this analysis, given the fact that most of the animals (Yorkshire boars) were the same in both analyses. However, had the Finnish Landrace been used as the validation population for the previous study, the significance of ASGA0070625 would not have been repeated and this SNP would have been claimed to be a population-specific marker for meat pH. Similarly, I199V was not repeated in the Yorkshire, raising a doubt that I199V is breed- or population-specific. But when haplotypes instead of single SNPs were used in the association analysis, the results were coherent: the same haplotype (HAP2) was significantly associated with pH in both breeds.

The haplotype with both *199I* and *24E* alleles (or *g.-157C* or *g.-58A*) was found favorable for pH measured from loin and ham in both breeds. This provides strong support for the hypothesis that allele *199I* alone does not create a positive effect on the pH level in muscle post mortem, but the animal has to carry an additional variation either in the promoter region of *PRKAG3* (*g.-157C* or *g.-58A* or both) or glutamate at amino acid position 24 (or 47 depending on the *PRKAG3* isoform used for naming). Analysis of the SNP effect on the protein function suggested that there may be some differences between transcripts. Based on a SIFT analysis [13], the K24E mutation showed a significant (SIFT score < 0.05) effect on the protein function in the ENSSSCT00000033825 transcript, but not in ENSSSCT00000017641 (Table 2). Thus the promoter SNPs may affect the expression of a specific transcript, and together with amino acid changes, may influence the function of *PRKAG3*. Interestingly, the haplotype with the lowest P-value and a positive association with meat quality in both of the studied breeds is similar to a wild boar haplotype, with the exception that the wild boars’ *199V* is replaced by *199I*.

## Conclusions

A single mutation in ASGA00070625, in the promoter region, in the amino acid at positions 24 or at 199 of *PRKAG3* is not alone sufficient to create a favorable effect on meat quality. Instead, a combination of variations or a haplotype with the promoter region variants *g.-157C* and *g.-58A* and amino acid substitutions *24E*, and *199I* of *PRKAG3* is needed to achieve a positive impact on meat quality traits, at least in the Finnish Yorkshire and Landrace populations. The results presented here can be directly applied in marker-assisted selection to improve the quality of pork meat.

## Methods

Animal material for this study included previously collected semen and hair samples of the boars thus no ethical approval was required. All phenotypic data were kindly supplied by the Figen Ltd (http://www.figen.fi).

### Animals and meat quality measurements

The study included 220 Finnish Yorkshire and 230 Finnish Landrace AI (artificial insemination) boars. Additionally, three European wild boars were sequenced, but no phenotypic observations were available for these boars. Breeding values of the studied boars were estimated using the full national pig registry data including meat quality measurements from several thousand animals. We used a single-trait BLUP procedure to estimate a breeding value for meat pH, color L* (lightness of meat) and a* (redness of meat). The statistical model included slaughter batch and sex as fixed, and litter and animal as random effects. The model was the same as used in national breeding value estimation in Finland, except that in the national evaluation all meat quality traits are analyzed simultaneously by a multitrait model, whereas in this study each trait was analyzed separately. We selected the single-trait approach to ensure that genetic correlation between traits did not affect the association results. The estimated breeding value (EBV) reflects the relative genetic merit of an animal. EBVs are generally more reliable than the animal’s own phenotype, because they are based on all available records on relatives and are simultaneously corrected for specific systematic and non-systematic effects specified in the estimation model. Most of the meat quality data for a specific AI boar is obtained from its progeny and its full- and half-sibs.

EBVs for meat quality traits are based on measurements taken from animals raised in a test station. Young piglets (on average 30 kg weight) are raised up to 100 kg live weight in a standardized test station environment. After the test period, all but the best young boars are sent to a slaughterhouse where pH and color L* and a* of meat are measured 24 h after slaughter. For this study, color L* and a* were measured on a freshly cut muscle surface with a Minolta CR 300 colorimeter and a CIELAB color scale standard [19,20], and pH was determined using a Knick 752 pH meter and an Ingold 406 electrode. Measurements were taken from loin (*longissimus*) and ham (*semimembranosus*) muscles. For more information on the measurement procedures, see Sevón-Aimonen et al. [1].

The studied Finnish Yorkshire and Landrace boars were born between 1992 and 2009, and included several relative pairs such as sire-son, full-sibs, grandsire-grandson, etc. Average relatedness between boars was 0.16 and 0.14 for the Yorkshire and Landrace, respectively.

### Genotyping and sequencing

Part of the SNP data presented in this study originate from our previous whole-genome analyses [11,14] using the PorcineSNP60 BeadChip (Illumina Ltd, San Diego, USA). Genotyping was performed at FIMM (Institute for Molecular Medicine Finland, Helsinki, Finland) or at GeneSeek (Lincoln, USA). DNA was extracted either from hair follicles or semen, with a target DNA concentration of 300 ng. SNPs were mapped to the pig genome build Sscrofa10.2. We restricted our statistical analysis to cover only SNPs located in a 20-Mb region surrounding *PRKAG3* (from 120 Mb to 140 Mb on chromosome 15), because our previous analyses had shown that the most significant SNPs for meat quality were in this region.

After designing primer pairs for genomic sequence analysis, we amplified the DNA fragments with gene-specific primers. PCR amplicons were purified using ExoSAP-IT™ (GE Healthcare, Piscataway, USA), and sequenced in both directions with the same primers as in the amplification procedures. Sequencing was performed on a 3500 × L Genetic Analyzer (Applied Biosystems, Carlsbad, USA) using a BigDye Terminator v3.1 kit (Applied Biosystems, Carlsbad, USA) and EtOH precipitation.

### Statistical method

Prior to association analysis, the EBVs were deregressed and their weights were calculated by the method proposed by Garrick et al. [21]. The method removes parent average effects on EBV, so that the deregressed EBV more closely reflects the animal’s own performance and the performance of its offspring. The deregression procedure also prevents regression towards the population mean, which is typical for EBVs which are based on a limited amount of information. Generally, the more reliable the deregressed EBV is, the more weight it receives in the association analysis.

Association analysis was performed either for individual SNPs or a combination of SNPs (haplotype). Each SNP/haplotype was analyzed separately for association with meat quality traits using the following mixed linear model:

$$y_{i}=\mu +b*x_{i}+a_{i}+e_{i},$$

where *y*_*i*_ is the deregressed EBV of the meat quality trait; *x*_*i*_ is the number of minor alleles (0, 1, or 2) of the tested SNP or the number of copies of the tested haplotype (0: an animal carries no copies; 1: an animal carries one copy; 2: an animal carries two copies); *b* is the corresponding regression coefficient; *a*_*i*_ is a random polygenic effect with a normal distribution with mean 0 and a variance-covariance structure of **A***σ*^*2*^_*a*_, where **A** is the additive relationship matrix and *σ*^*2*^_*a*_ is the polygenic variance; and *e*_*i*_ is a random residual effect with a normal distribution with mean 0 and a variance-covariance structure of **I***σ*^*2*^_*e*_*/w*_*i*_ , where **I** is an identity matrix, *σ*^*2*^_*e*_ is the residual variance, and *w*_*i*_ is the weight. Association analyses were performed using the AI-REML method in the DMU program package [22]. Haplotypes were estimated with FastPHASE [23], and linkage disequilibrium plots were produced with Haploview [24].

## Abbreviations

AI: Artificial insemination; AMPK: AMP-activated protein kinase; EBV: Estimated breeding value; LD: Linkage disequilibrium.

## Competing interests

The authors declare that they have no competing interests.

## Authors’ contributions

PU carried out the data analysis and drafted the manuscript. AS performed the sequencing and SNP calling, and helped to draft the manuscript. Both authors read and approved the final manuscript.

## Authors’ information

PU: current address: Department of Agricultural Sciences, Animal Breeding, FI-00014 University of Helsinki, Finland; AS: current address: MTT Agrifood Research Finland, Biotechnology and Food Research, FI-31600 Jokioinen, Finland.
